# Supplementary material for: Deciphering cell–cell communication at single-cell resolution for spatial transcriptomics with subgraph-based graph attention network
Source: Nat Commun. 2024 Aug 18;15:7101. doi: 10.1038/s41467-024-51329-2 (PMC11330978; doi:10.1038/s41467-024-51329-2)
Supplement: Supplementary file 1 — Supplementary Figs. [file 41467_2024_51329_MOESM1_ESM.pdf]

Supplementary Figures

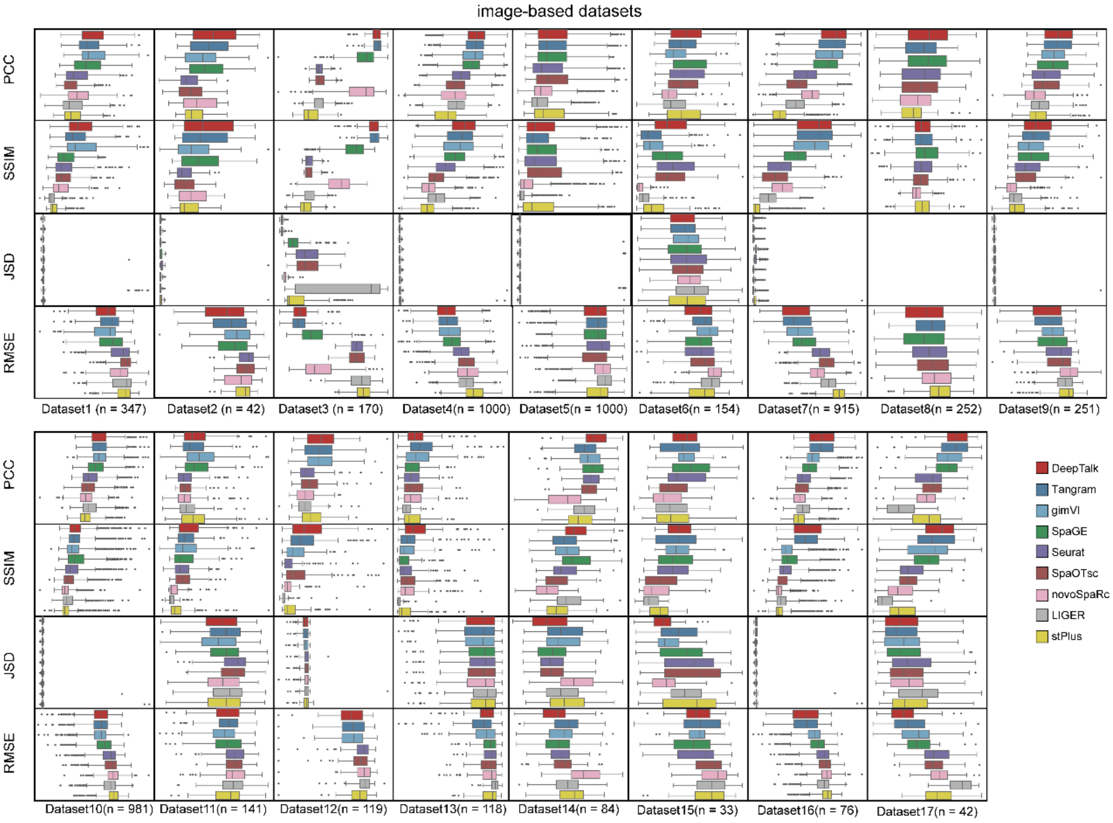

Supplementary Fig. 1: The Boxplots of PCC, SSIM, RMSE, JSD values of each integration method in predicting the spatial distribution of RNA transcripts of image-based datasets. The boxplot of PCC, SSIM, RMSE, JSD values of each integration method in predicting the spatial distribution of RNA transcripts of 17 image-based paired spatial transcriptomics and scRNA-seq datasets. Center line, median; box limits, upper and lower quartiles; whiskers, 1.5× interquartile range, individual outliers are denoted by separate dots.; the number of genes for each dataset is shown at the top of each panel. Source data are provided as a Source Data file.

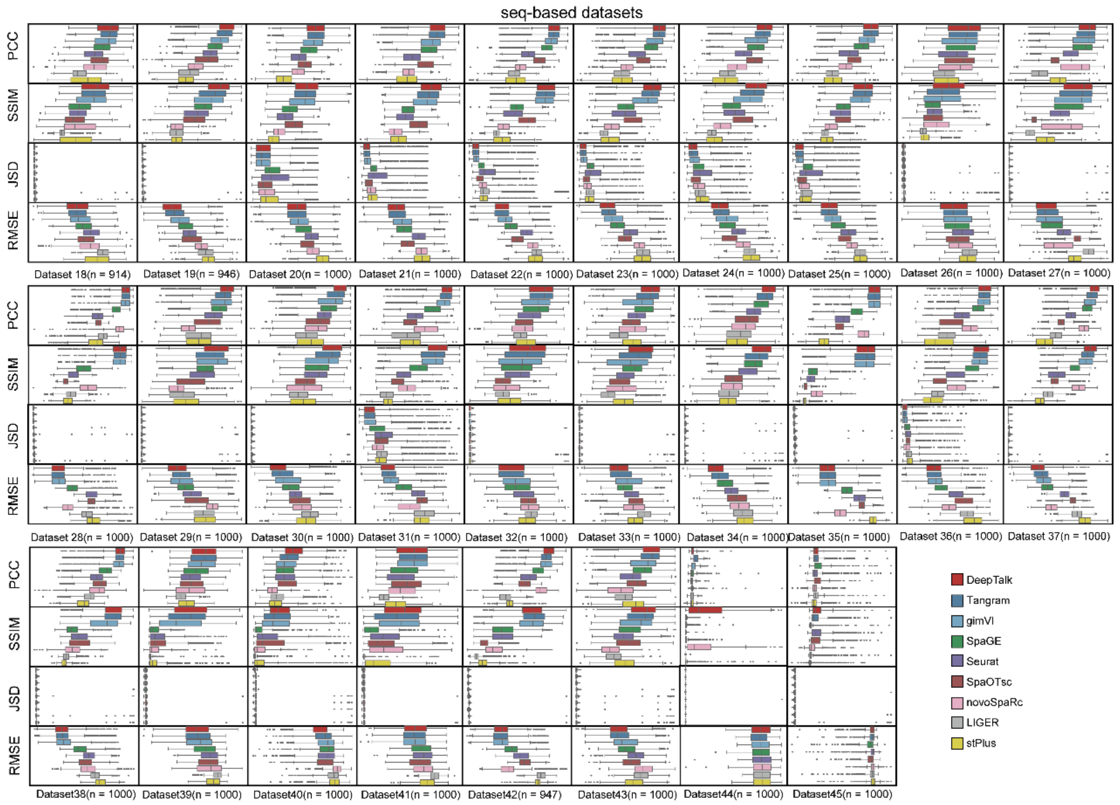

Supplementary Fig. 2: The Boxplots of PCC, SSIM, RMSE, JSD values of each integration method in predicting the spatial distribution of RNA transcripts of seq-based datasets.

The boxplot of PCC, SSIM, RMSE, JSD values of each integration method in predicting the spatial distribution of RNA transcripts of 28 seq-based paired spatial transcriptomics and scRNA-seq datasets. Center line, median; box limits, upper and lower quartiles; whiskers, 1.5× interquartile range, individual outliers are denoted by separate dots; the number of genes for each dataset is shown at the top of each panel. Source data are provided as a Source Data file.

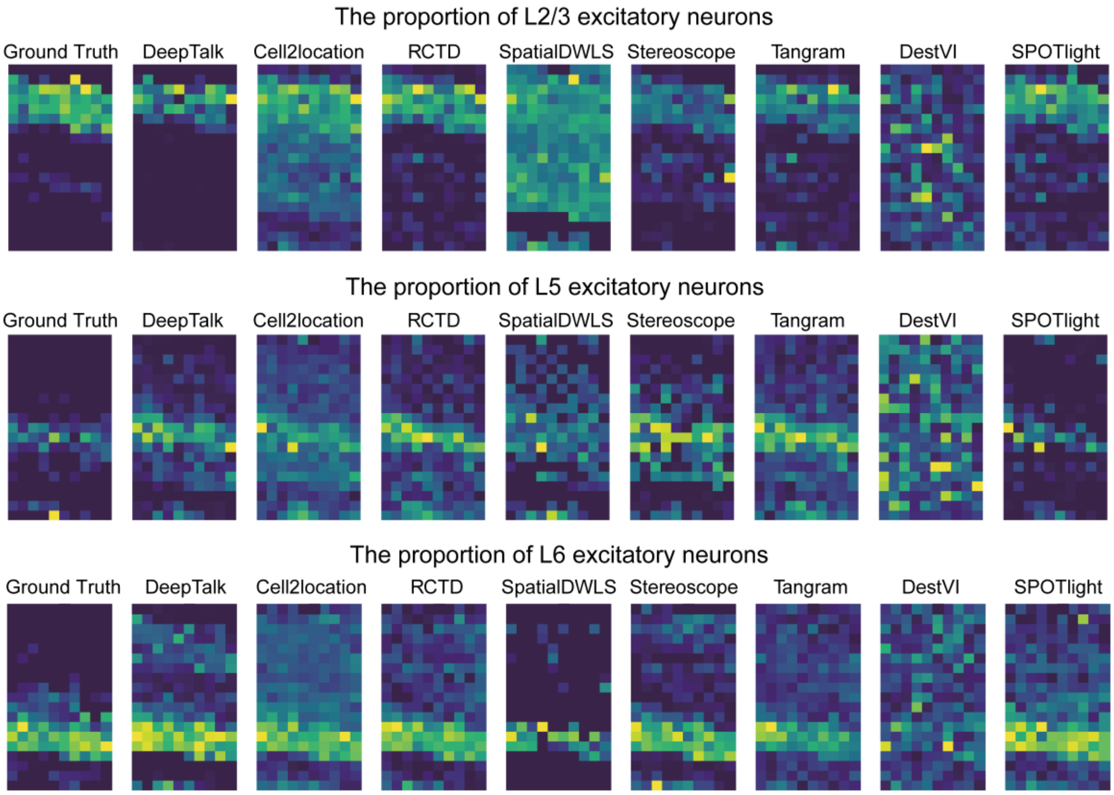

Supplementary Fig. 3: The proportion of L2/3, L5 and L6 excitatory neurons in the spots simulated from STARmap dataset.

The proportion of L2/3, L5 and L6 excitatory neurons in the spots simulated from STARmap dataset, including the ground truth and the predicted results of 8 integration methods.

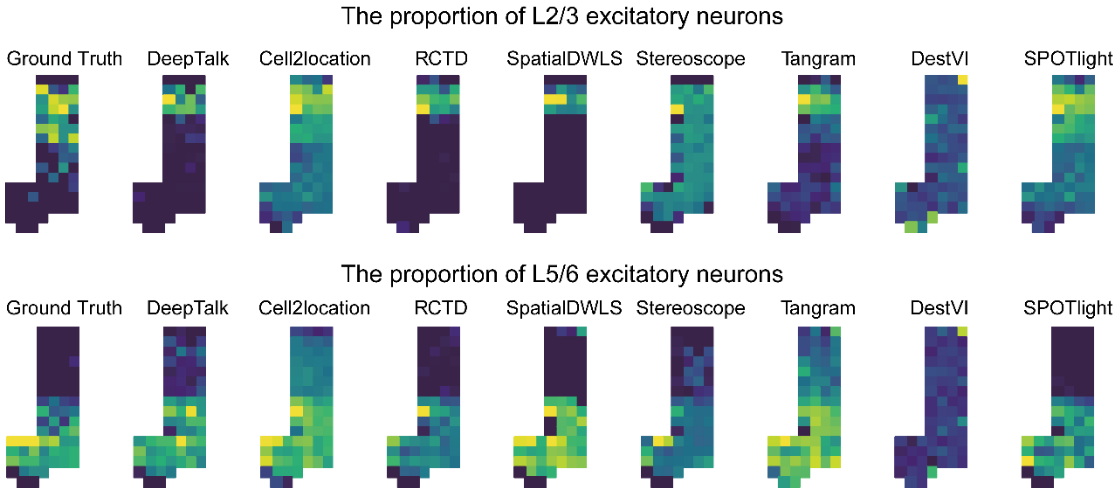

Supplementary Fig. 4: The proportion of L2/3, L5/6 excitatory neurons in the spots simulated from seqFISH+ dataset.

The proportion of L2/3, L5/6 excitatory neurons in the spots simulated from seqFISH+ dataset, including the ground truth and the predicted results of 8 integration methods.

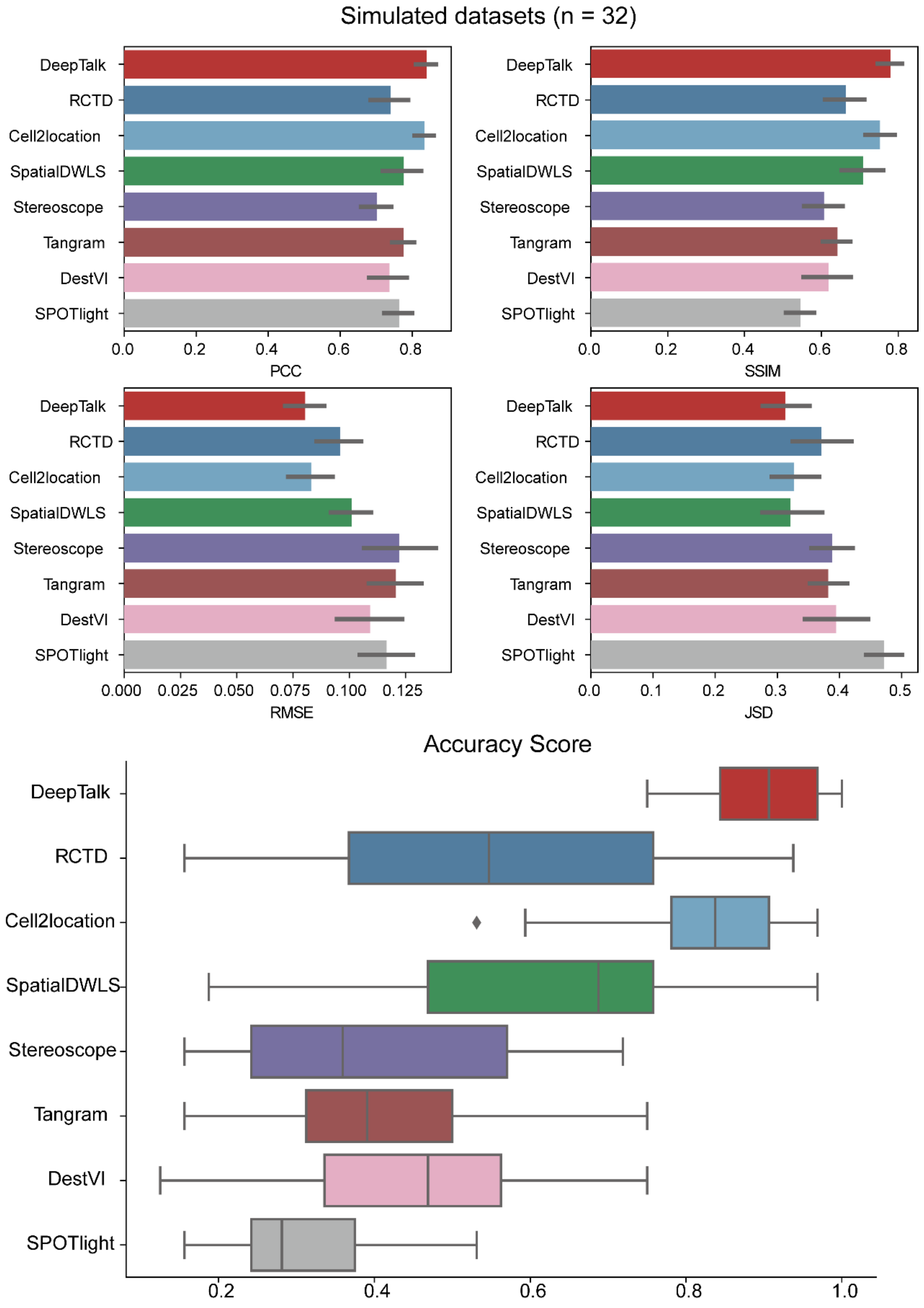

Supplementary Fig. 5: Bar plots of PCC, SSIM, RMSE, and JSD of 8 integration method for 32 simulated datasets and Boxplots of AS of the 8 integration methods for all the 32 simulated datasets. Center line, median; box limits, upper and lower quartiles; whiskers, 1.5× interquartile range, individual outliers are denoted by separate dots; n = 32 independent datasets. Source data are provided as a Source Data file.

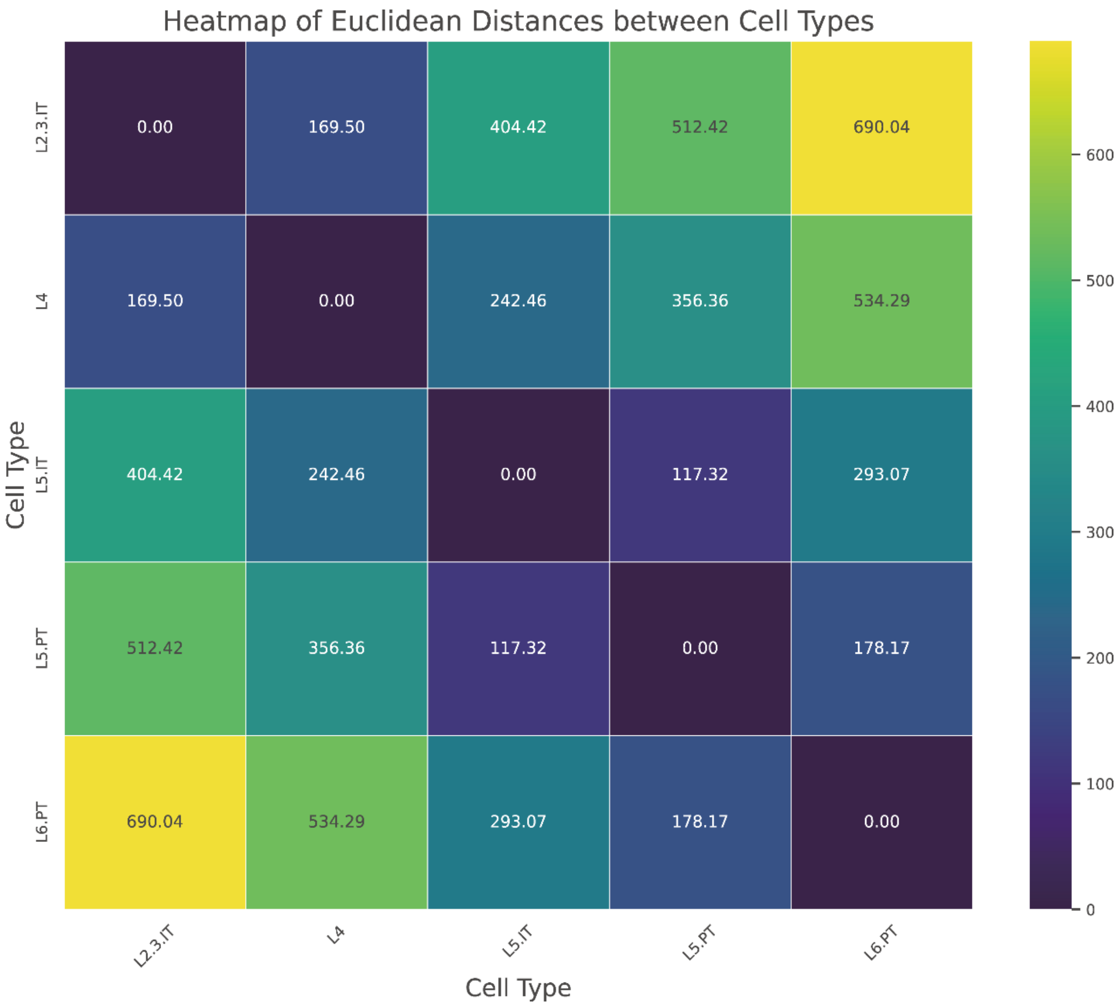

Supplementary Fig. 6: The distance between five cell types for MERFISH data of mouse visual cortex. The plot of distance between 5 cell types for MERFISH dataset of mouse visual cortex.

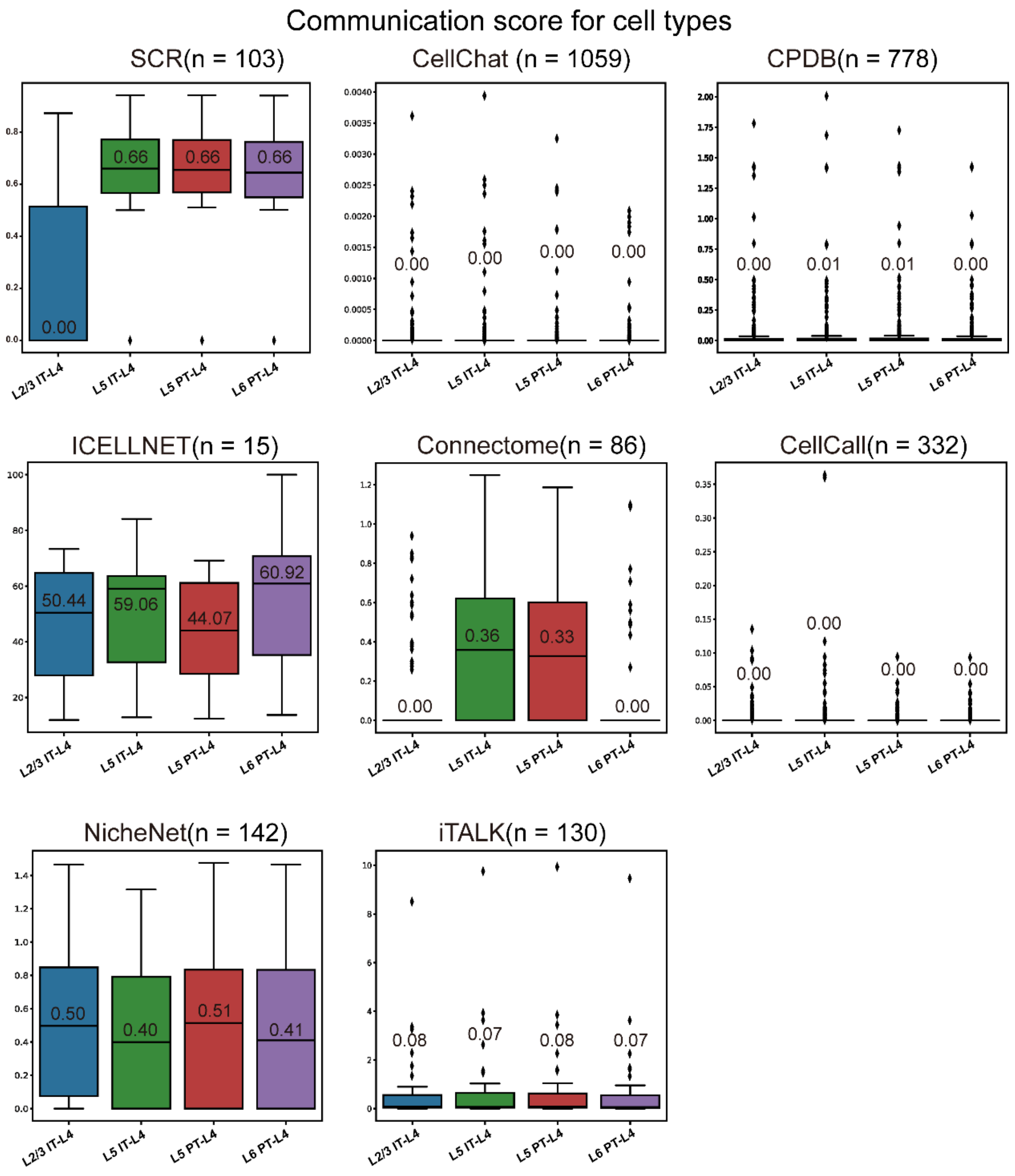

Supplementary Fig. 7: Comparison of the cell-cell communication score between spatially adjacent and distant cell types for 8 scRNA-seq based tools. This graph compares the communication scores between L4 cells and other cell types, including L2/3 IT, L5 IT, L5 PT, and L6 PT, highlighting the differences based on spatial proximity. Center line, median; box limits, upper and lower quartiles; whiskers, 1.5× interquartile range, individual outliers are denoted by separate dots; the number of predicted L-R pairs is shown at the top of each panel. Source data are provided as a Source Data file.

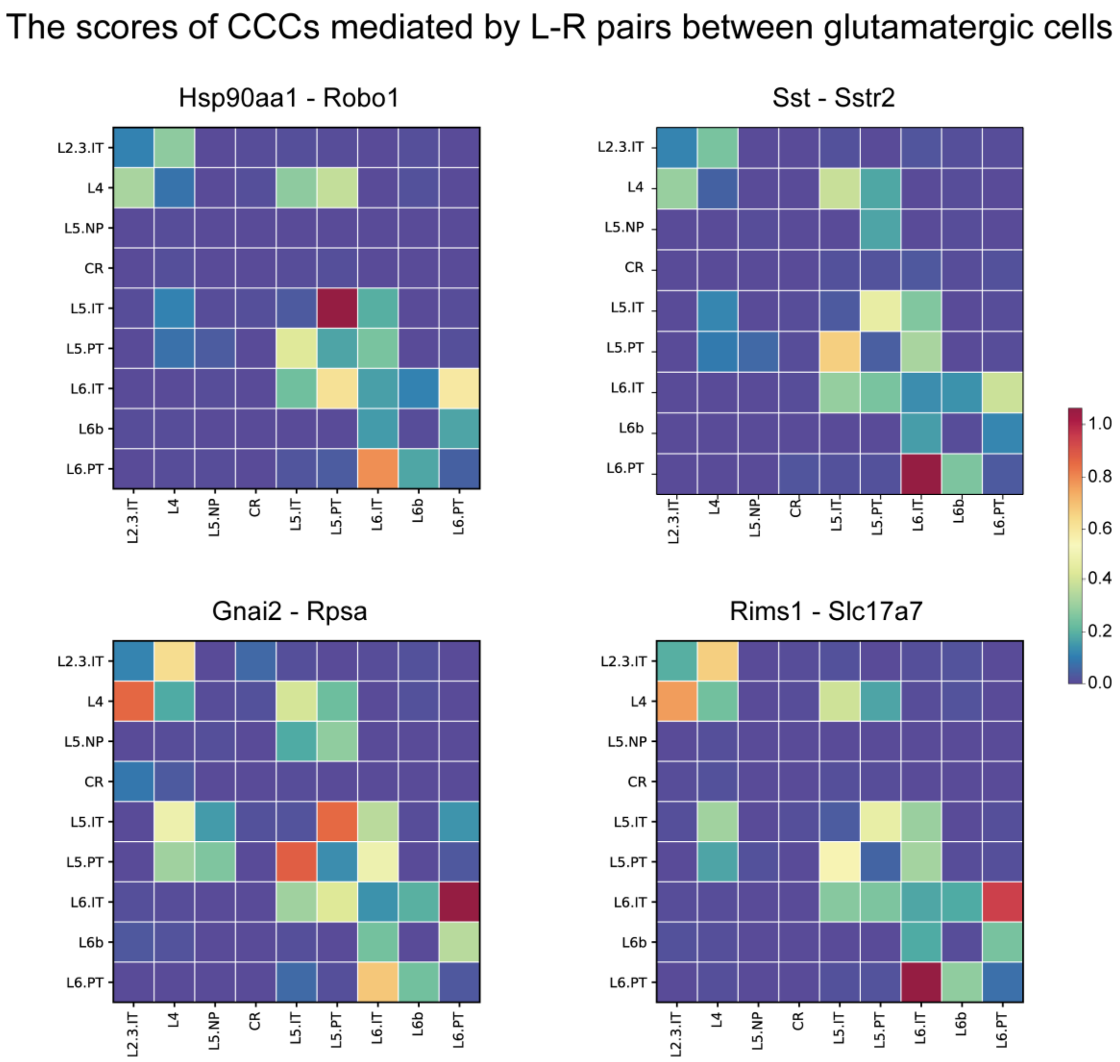

Supplementary Fig. 8: The scores of CCCs mediated by L-R pairs between glutamatergic cells on MERFISH data. The predicted CCCs mediated by L-R pairs. The scores of CCCs mediated by L-R pairs between glutamatergic cells.

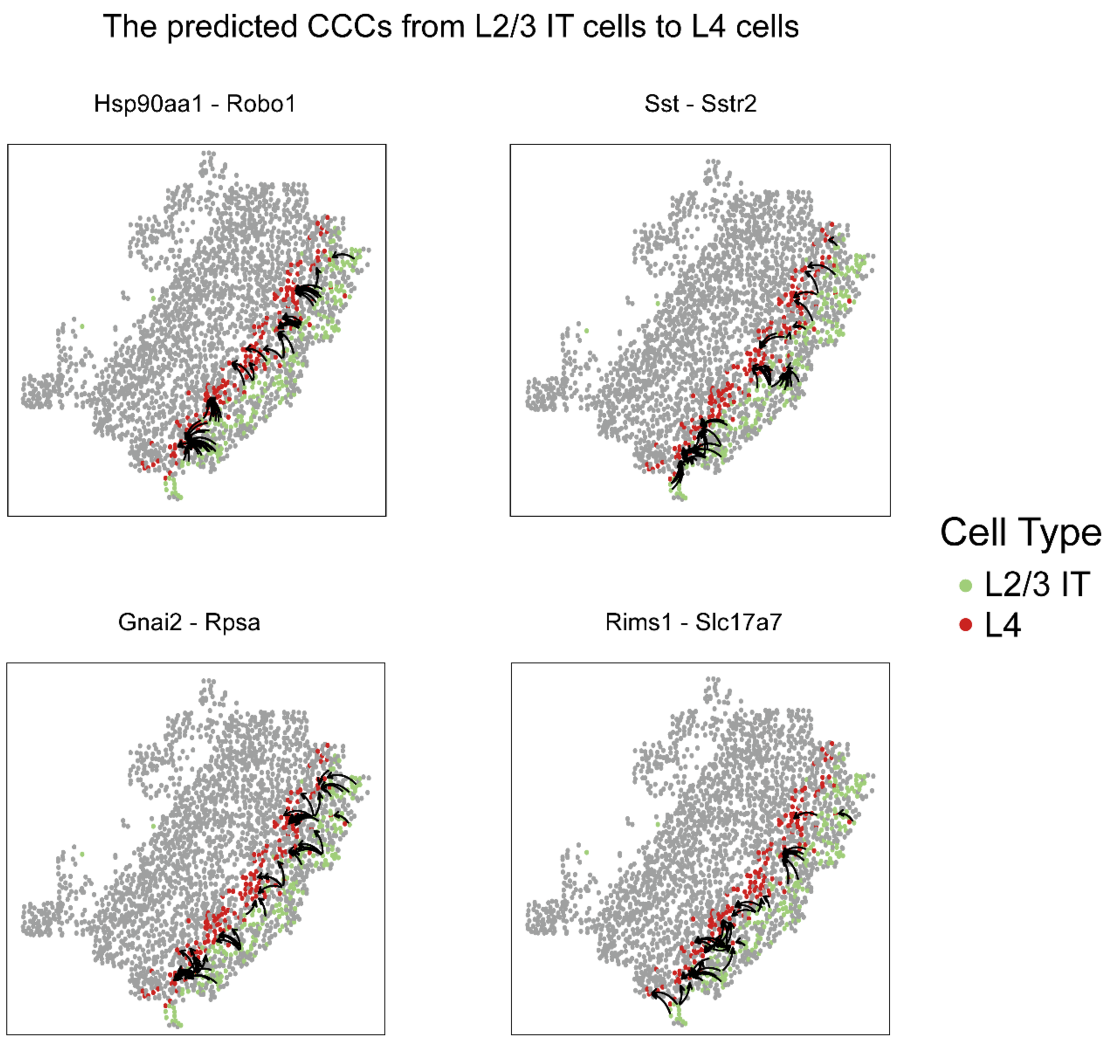

Supplementary Fig. 9: The predicted CCCs from L2/3 IT cells to L4 cells on MERFISH data. The predicted CCCs from L2/3 IT cells to L4 cells mediated by L-R pairs.

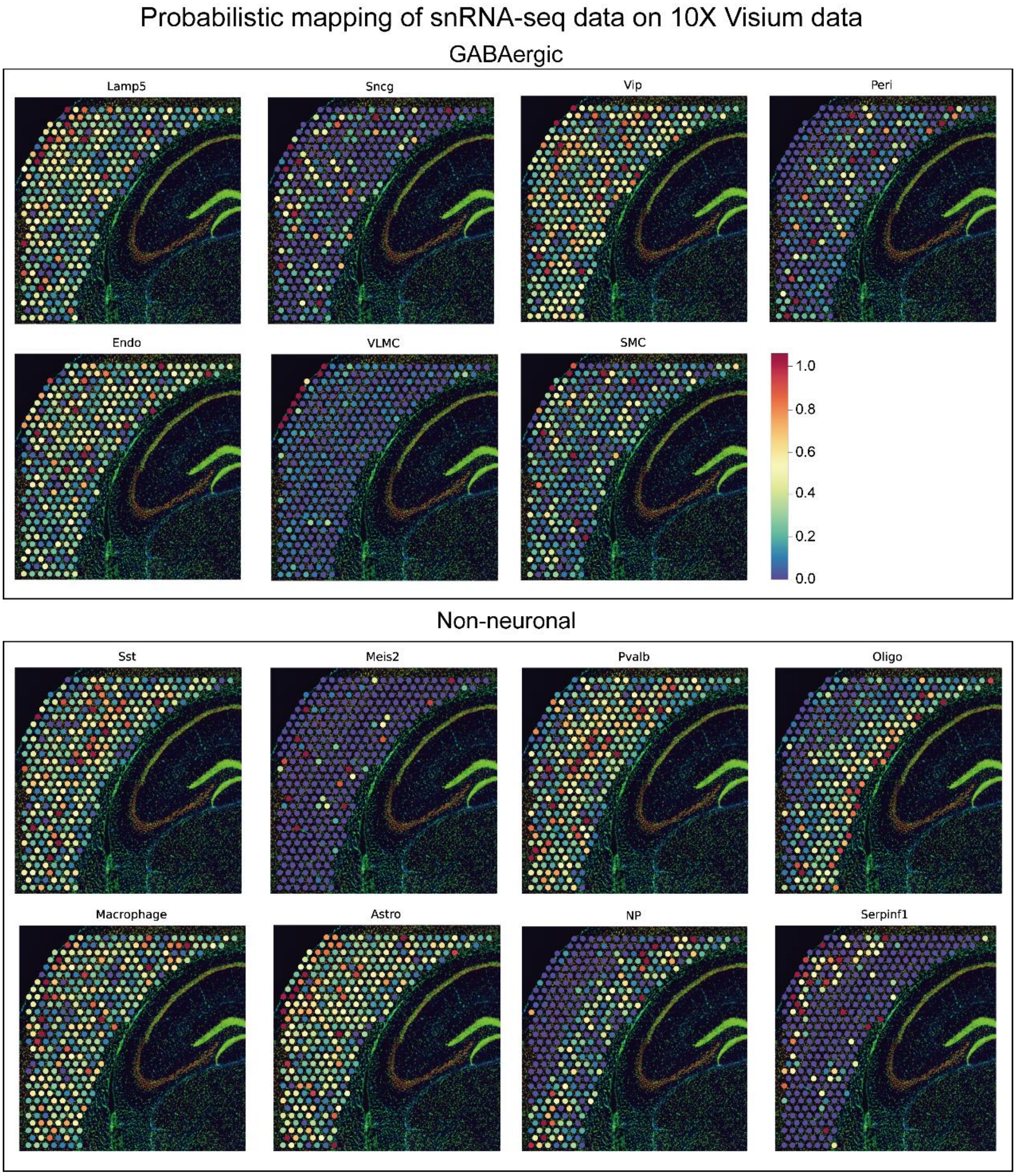

Supplementary Fig. 10: Probabilistic mapping of scRNA-seq data on 10X Visium data. Probability of mapping (color bar) of each cell subset in each of three major categories.

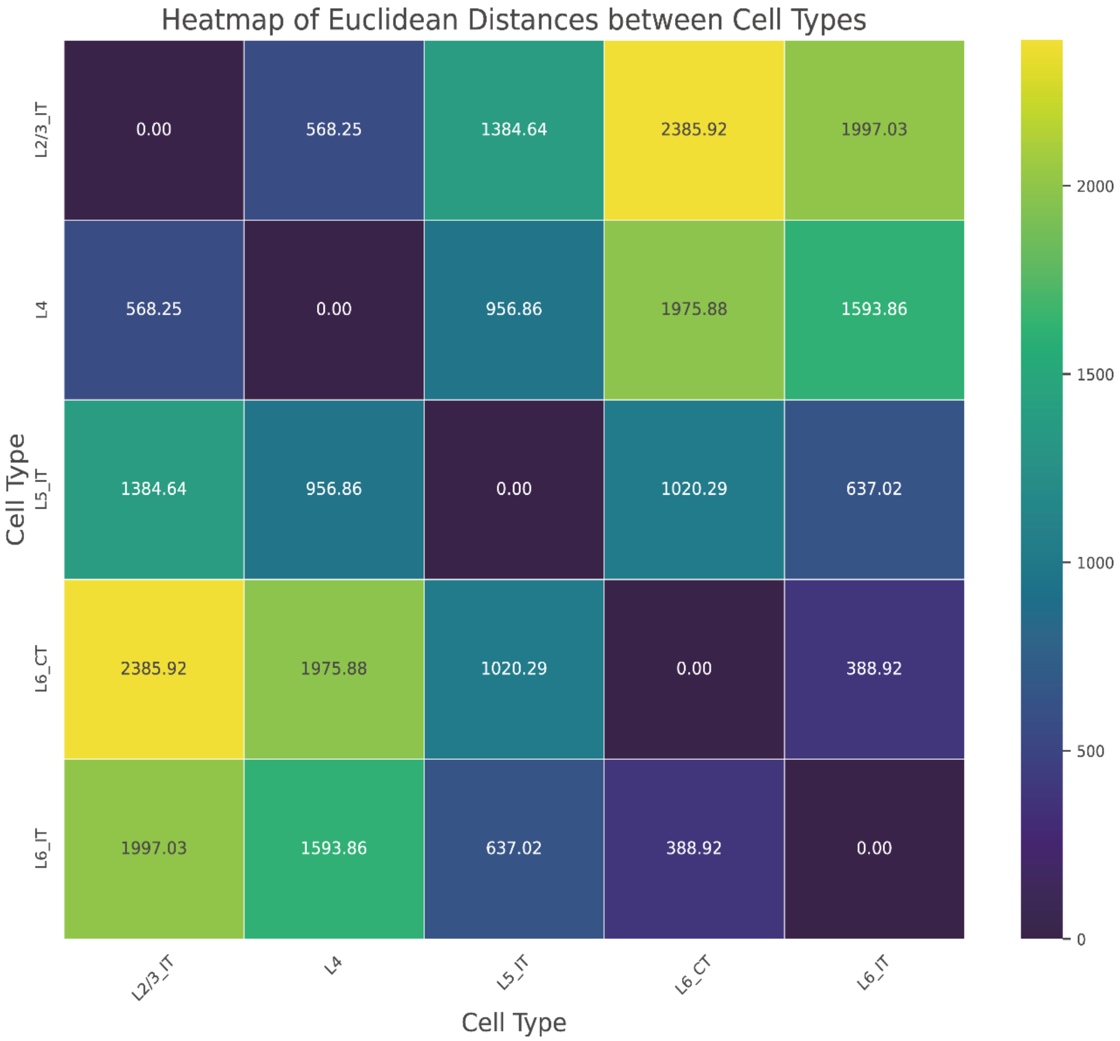

Supplementary Fig. 11: The distance between five cell types for 10X Visium data of adult mouse. The plot of distance between 5 cell types for MERFISH dataset of 10X Visium data.

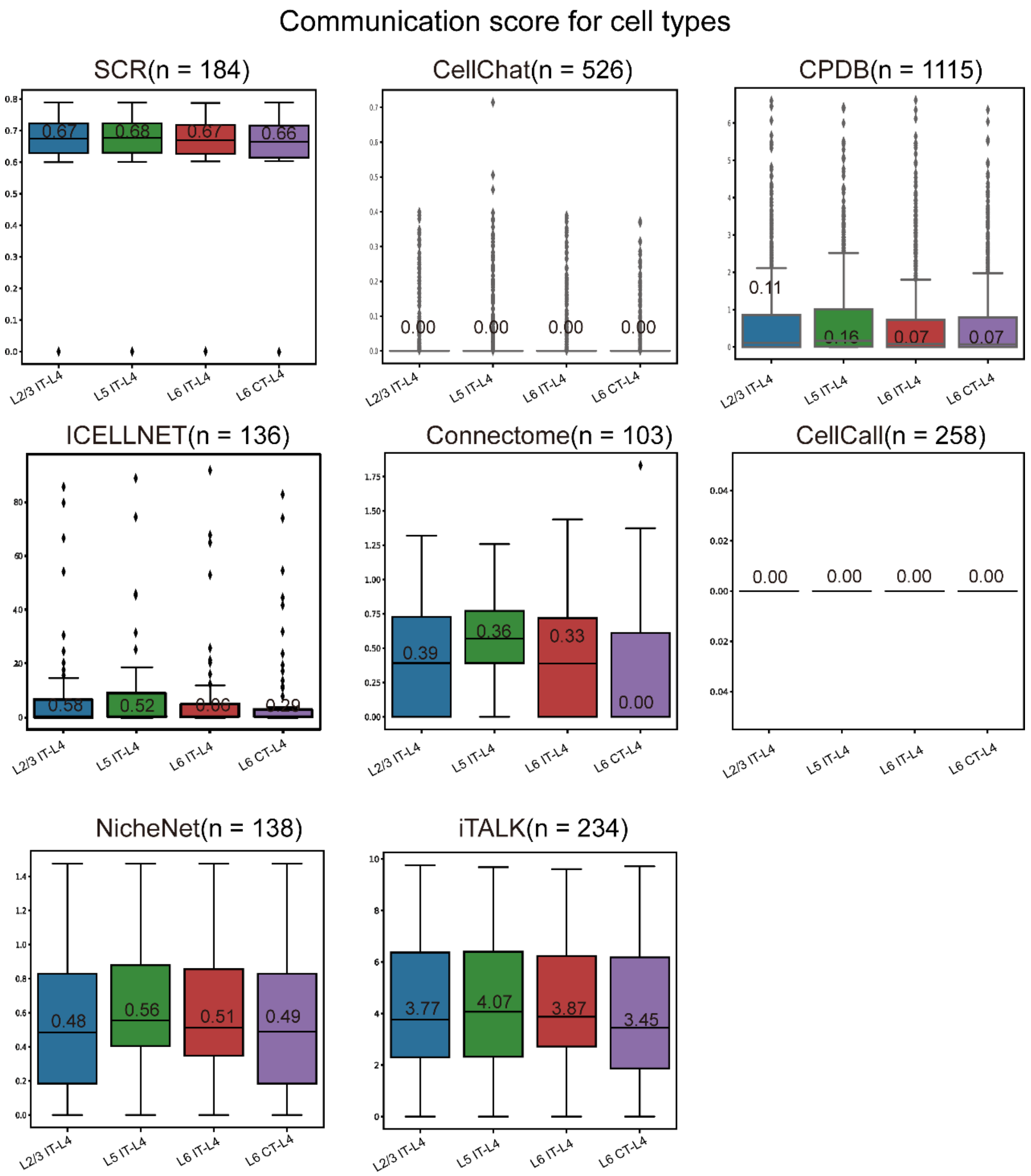

Supplementary Fig. 12: Comparison of the cell-cell communication score between spatially adjacent and distant cell types for 8 scRNA-seq based tools. This graph compares the communication scores between L4 cells and other cell types, including L2/3 IT, L4, L5 IT, L6 IT, and L6 CT, highlighting the differences based on spatial proximity. Center line, median; box limits, upper and lower quartiles; whiskers, 1.5× interquartile range, individual outliers are denoted by separate dots; the number of predicted L-R pairs is shown at the top of each panel. Source data are provided as a Source Data file.

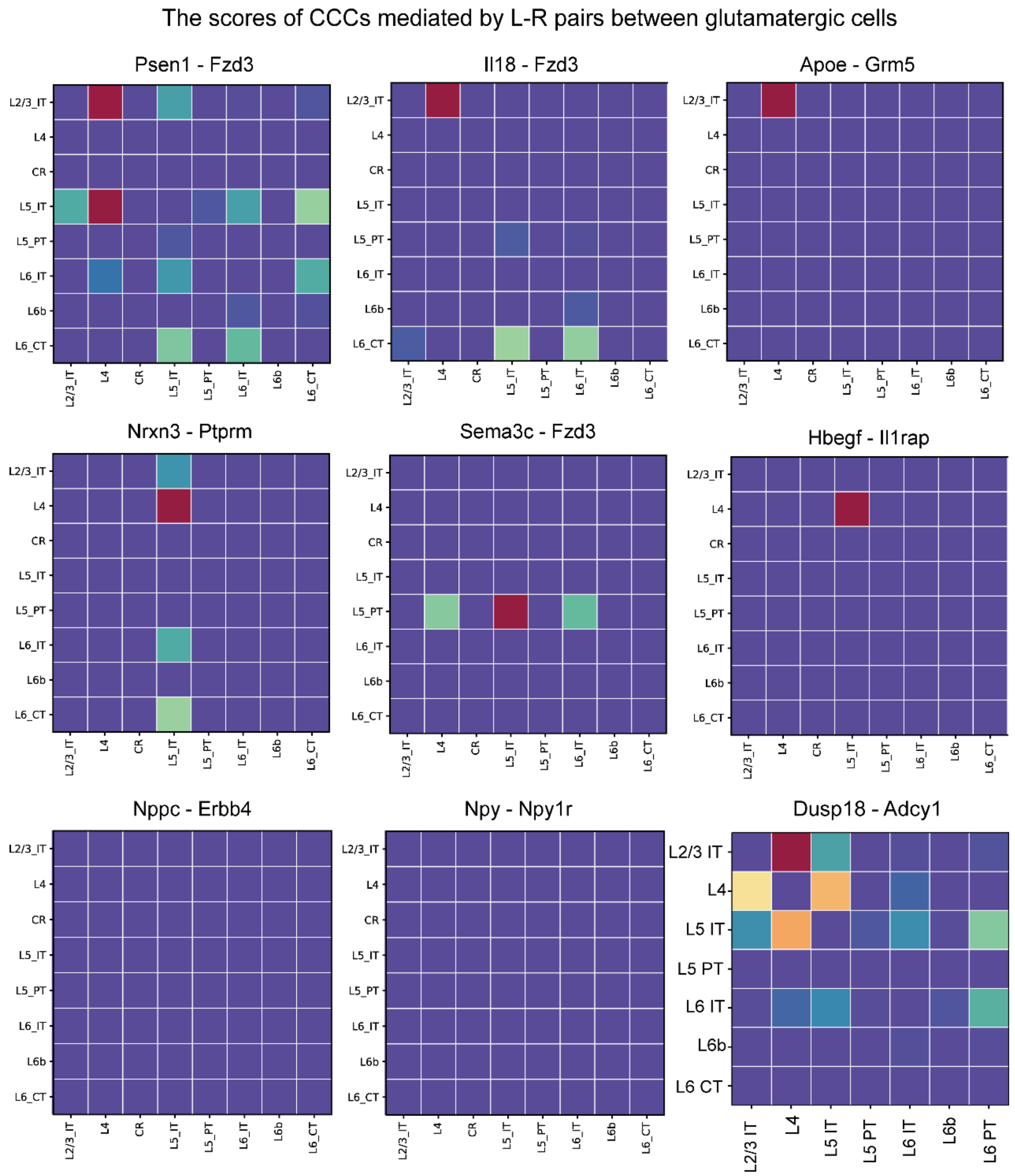

Supplementary Fig. 13: The scores of CCCs mediated by L-R pairs between glutamatergic cells on 10X Visium data. The predicted CCCs mediated by L-R pairs. The scores of CCCs mediated by L-R pairs between glutamatergic cells.

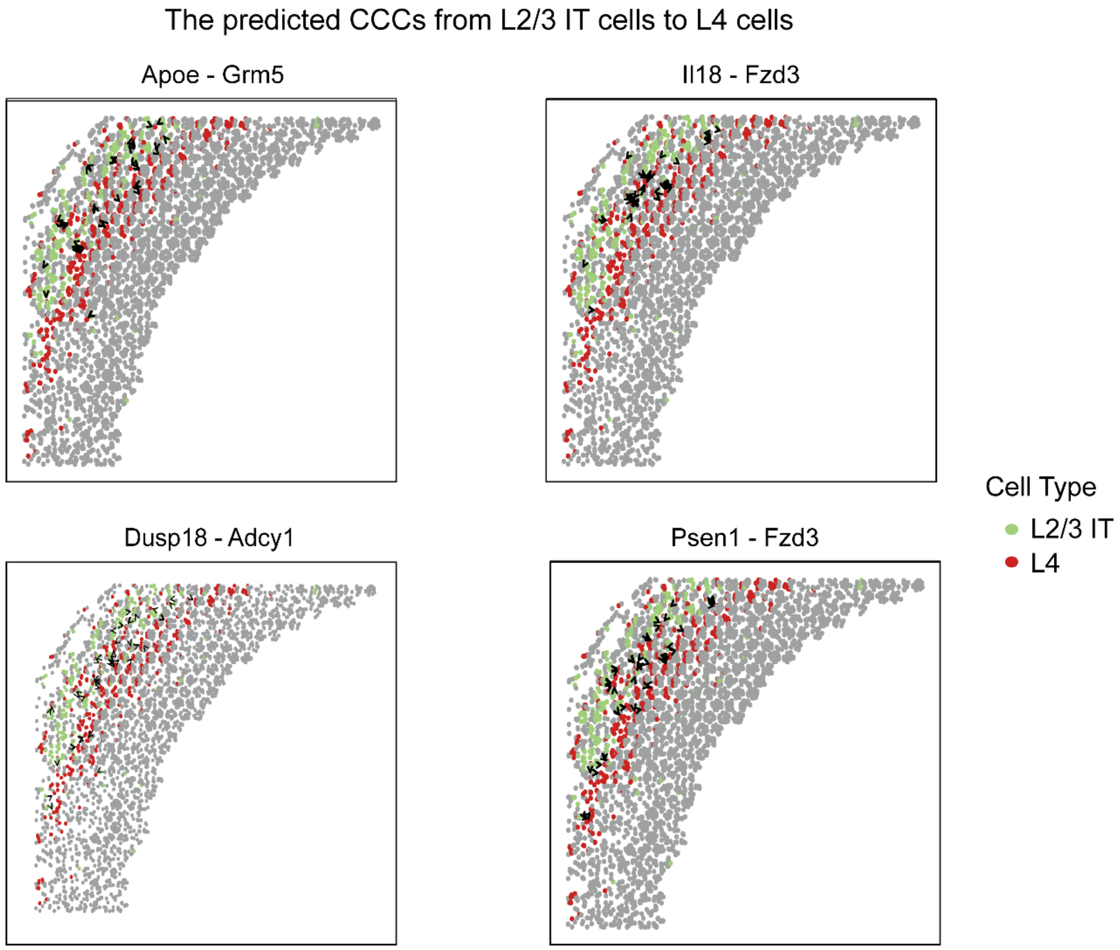

Supplementary Fig. 14: The predicted CCCs from L2/3 IT cells to L4 cells on 10X Visium data. The predicted CCCs from L2/3 IT cells to L4 cells mediated by L-R pairs.

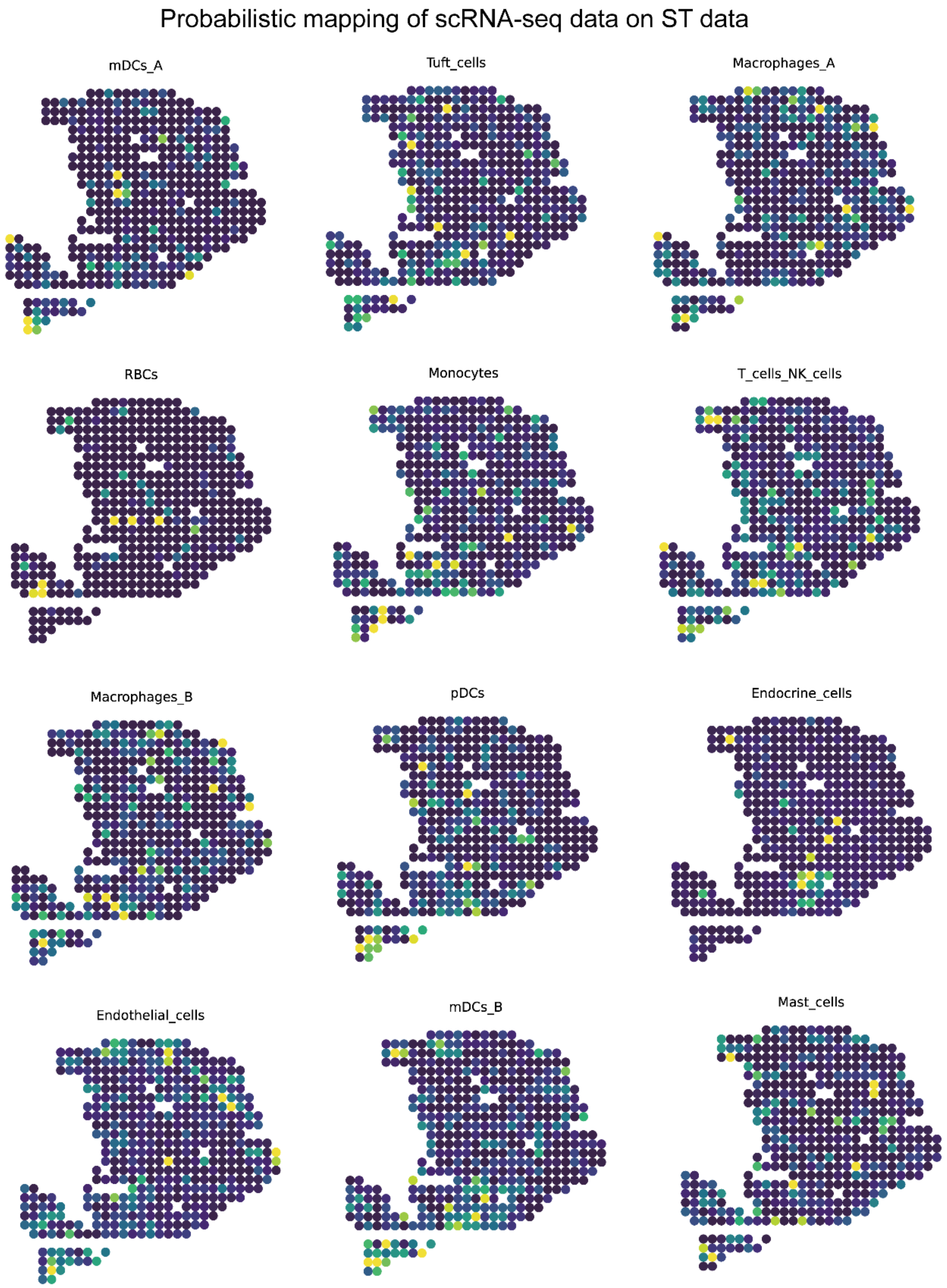

Supplementary Fig. 15: Probabilistic mapping of scRNA-seq data on ST data. Probability of mapping of each cell subset in each of three major categories.

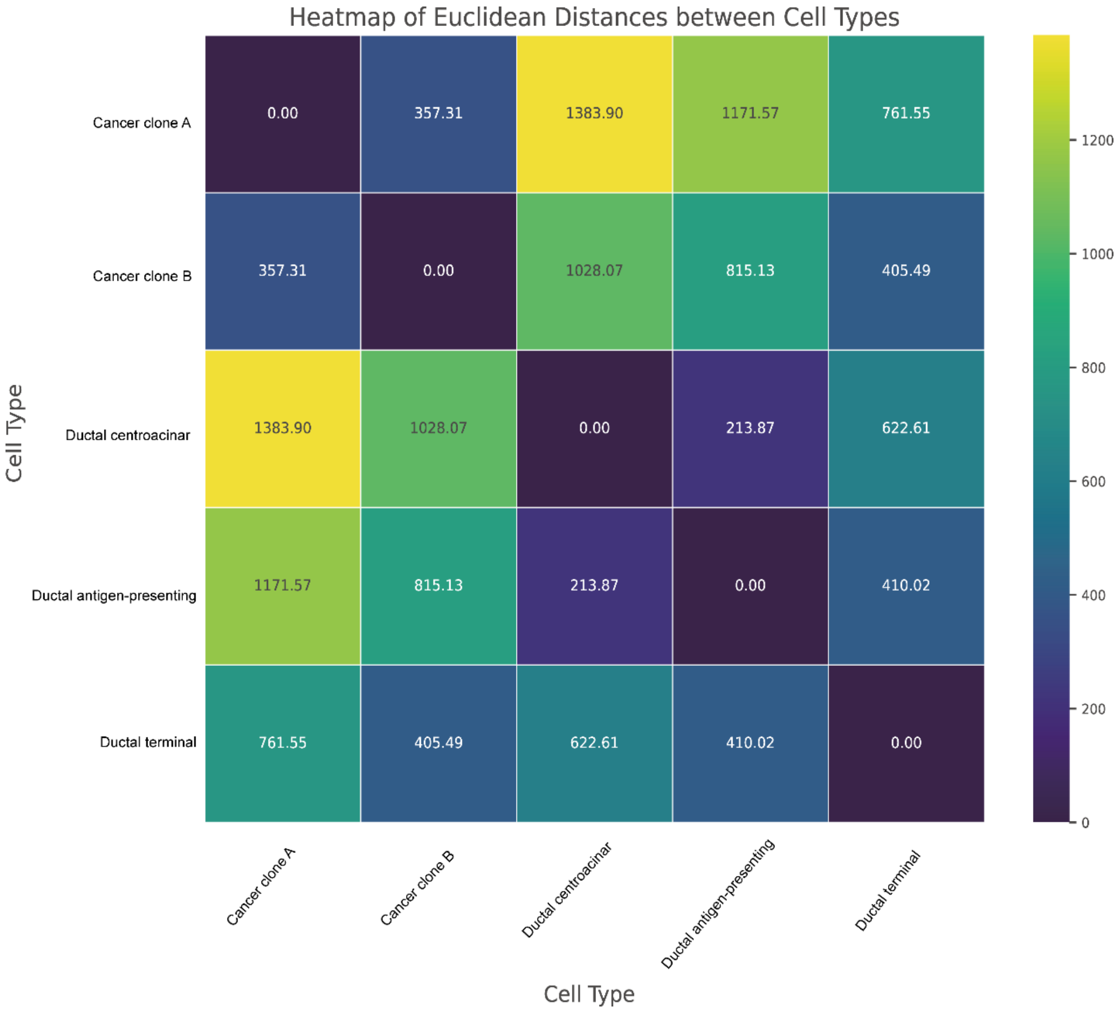

Supplementary Fig. 16: The distance between five cell types for ST data of adult mouse. The plot of distance between 5 cell types for MERFISH dataset of ST data.

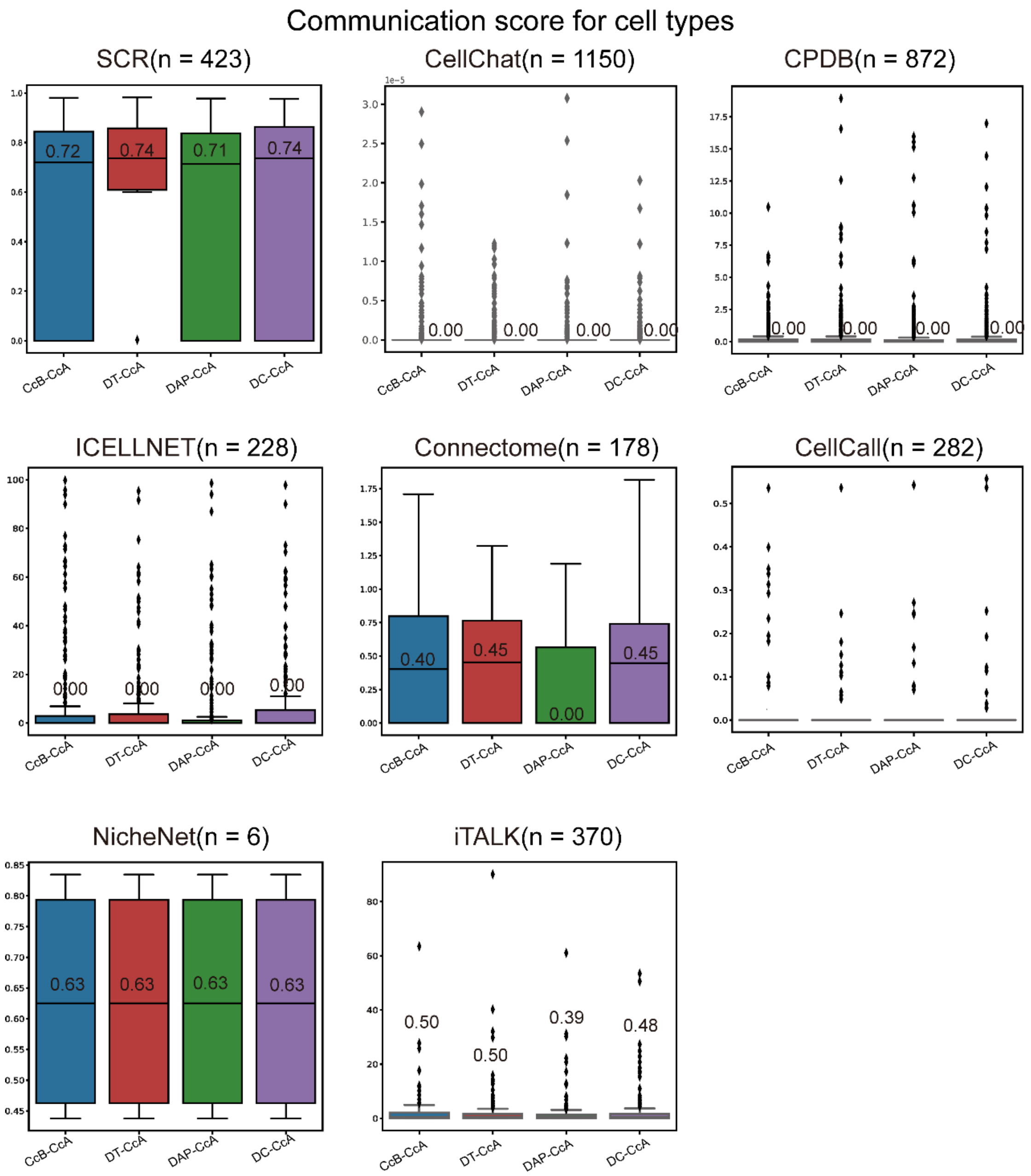

Supplementary Fig. 17: Comparison of the cell-cell communication score between spatially adjacent and distant cell types for 8 scRNA-seq based tools. This graph compares the communication scores between Cancer clone A (CCA) cells and other cell types, including Cancer clone B (CCB), Ductal terminal (DT), Ductal antigen-presenting (DAP), and Ductal centroacinar (DC), highlighting the differences based on spatial proximity. Center line, median; box limits, upper and lower quartiles; whiskers, 1.5× interquartile range, individual outliers are denoted by separate dots; the number of predicted L-R pairs is shown at the top of each panel. Source data are provided as a Source Data file.

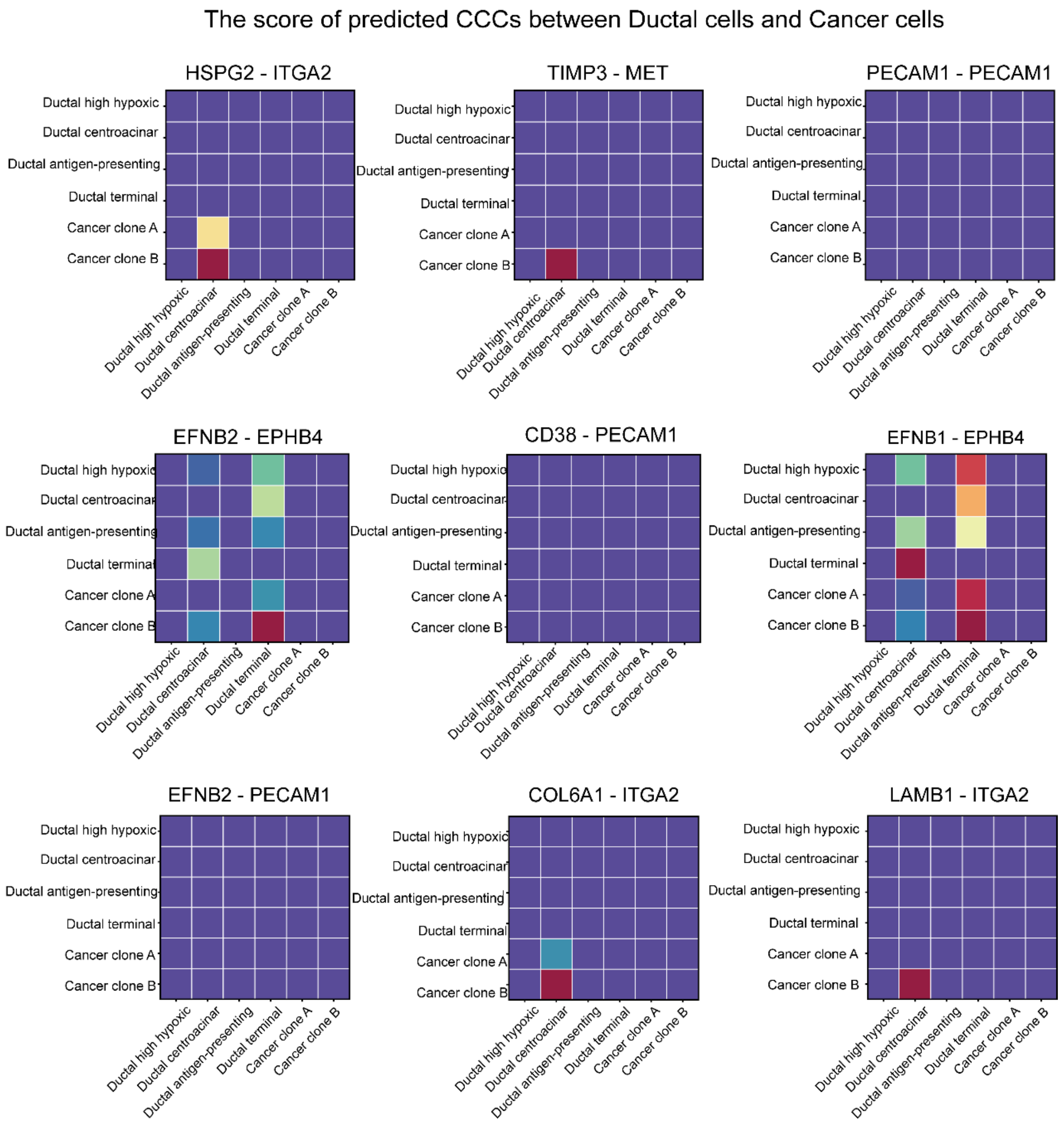

Supplementary Fig. 18: The scores of CCCs mediated by L-R pairs between Ductal cells and Cancer cells on ST data.

The predicted CCCs mediated by L-R pairs. The scores of CCCs mediated by L-R pairs between Ductal cells and Cancer cells.

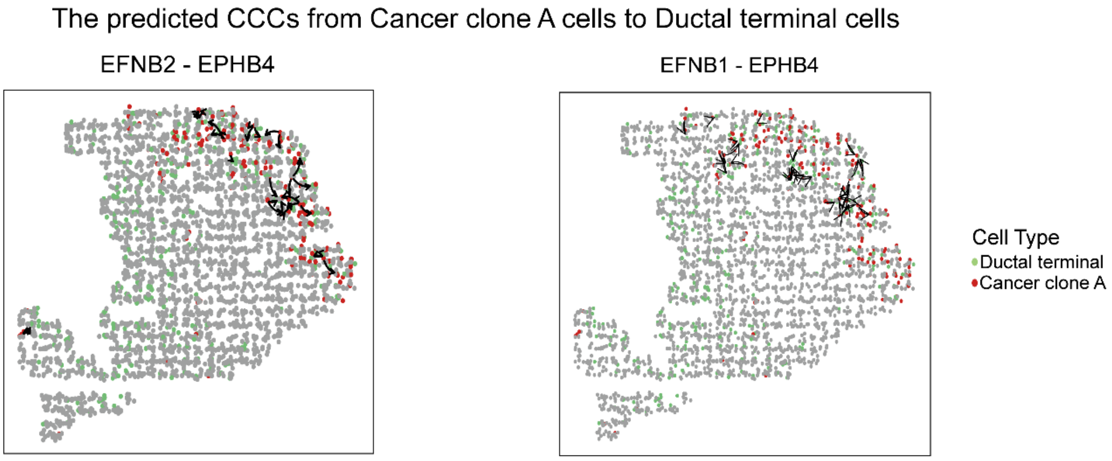

Supplementary Fig. 19: The predicted CCCs from from Cancer clone A cells to Ductal terminal cells on ST data.

The predicted CCCs from Cancer clone A cells to Ductal terminal cells mediated by EFNB2-EPHB4 pairs

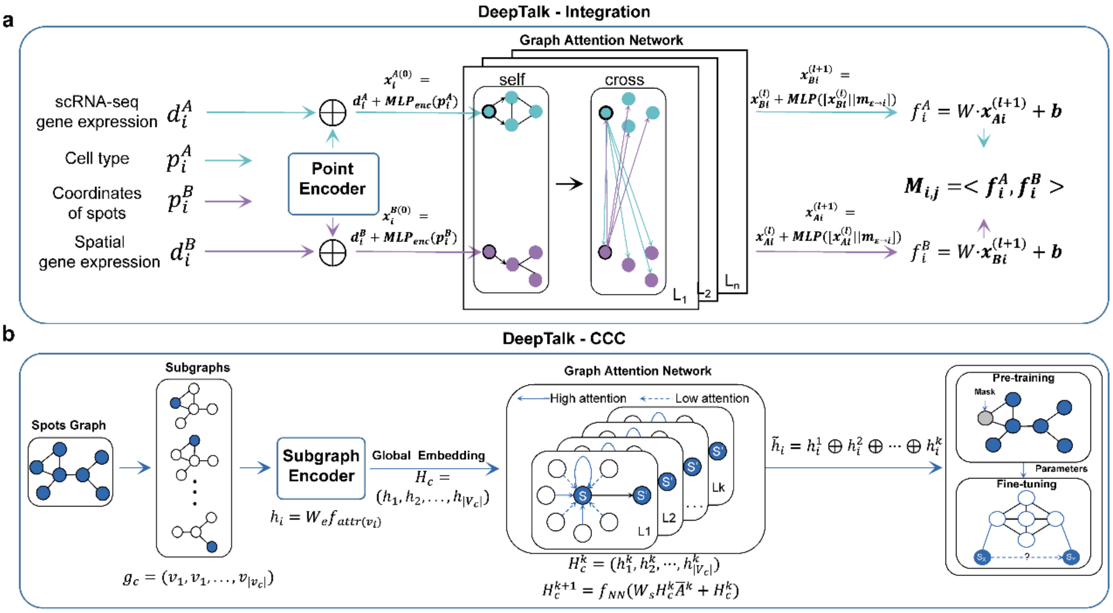

Supplementary Fig. 20: The DeepTalk Framework.

a. Utilizing a sophisticated Graph Neural Network (GNN) with attention mechanisms, we've seamlessly merged single-cell RNA sequencing (scRNA-seq) and spatial transcriptomics (ST) data. This GNN excels in creating matching descriptors by promoting feature exchange among initial data attributes. We've carefully designed a node encoder to transform cell locations into precise vectors. These, along with transcriptomic data, are then merged using a Multi-Layer Perceptron (MLP), embedding them into rich, high-dimensional representations. This enables the GNN to simultaneously harness both types of data during subsequent inference. We construct a graph combining transcriptomic nodes with cell locations, using self-loops and cross-edges for intra- and cross-omics connections, respectively. Messages are effectively propagated along these edges using a specific equation. Each node iteratively updates its representation by aggregating messages from all graph edges. An attention mechanism, similar to database retrieval, aids message aggregation, with self- and cross-attention for various edge types. The obtained matching descriptors are not normalized, reflecting prediction confidence. A similarity matrix captures descriptor similarities, facilitating effective pairwise scoring. **b.** Based on integrated cellular and spatial data, we've constructed a cellular graph where nodes are cells and edges represent relationships. During pre-training, a random walk strategy creates subgraphs for each cell, labeled as GC. These subgraphs capture the graph's connectivity patterns. Distinct subgraphs, denoted as gc, are produced for each node during pre-training and fine-tuning. These consist of low-dimensional vector representations derived by mapping node attributes and structural embeddings. The main objective is to transform global embeddings into representations reflecting nodes' prominent roles within the gc framework. This is achieved through transformation layers scrutinizing high-order relationships. A semantic affinity matrix, an asymmetric weight matrix, underscores the varying impacts cells can have on each other. Within each graph attention network, this matrix undergoes iterative learning by evaluating node connectivity. Embeddings are refined through message dissemination within the subgraph. Multiple graph attention networks formulate numerous embeddings for each node, which are aggregated into contextual embeddings for prediction endeavors. During pre-training, a linear projection function forecasts concealed nodes' likelihood, while during fine-tuning, a singular-layer feed-forward network with a softmax activation is used for binary link prediction.
