# Supplementary material for: Deciphering cell–cell communication at single-cell resolution for spatial transcriptomics with subgraph-based graph attention network
Source: Nat Commun. 2024 Aug 18;15:7101. doi: 10.1038/s41467-024-51329-2 (PMC11330978; doi:10.1038/s41467-024-51329-2)
Supplement: Supplementary file 3 — Reporting Summary [file 41467_2024_51329_MOESM3_ESM.pdf]

nature portfolio

Corresponding author(s): Qinghua Jiang

Last updated by author(s): Jul 11, 2024

Reporting Summary

Nature Portfolio wishes to improve the reproducibility of the work that we publish. This form provides structure for consistency and transparency in reporting. For further information on Nature Portfolio policies, see our Editorial Policies and the Editorial Policy Checklist.

Statistics

For all statistical analyses, confirm that the following items are present in the figure legend, table legend, main text, or Methods section.

n/a	Confirmed	
☐	☒	The exact sample size (*n*) for each experimental group/condition, given as a discrete number and unit of measurement
☐	☒	A statement on whether measurements were taken from distinct samples or whether the same sample was measured repeatedly
☐	☒	The statistical test(s) used AND whether they are one- or two-sided<br>*Only common tests should be described solely by name; describe more complex techniques in the Methods section.*
☒	☐	A description of all covariates tested
☐	☒	A description of any assumptions or corrections, such as tests of normality and adjustment for multiple comparisons
☐	☒	A full description of the statistical parameters including central tendency (e.g. means) or other basic estimates (e.g. regression coefficient) AND variation (e.g. standard deviation) or associated estimates of uncertainty (e.g. confidence intervals)
☐	☒	For null hypothesis testing, the test statistic (e.g. *F*, *t*, *r*) with confidence intervals, effect sizes, degrees of freedom and *P* value noted<br>*Give P values as exact values whenever suitable.*
☒	☐	For Bayesian analysis, information on the choice of priors and Markov chain Monte Carlo settings
☐	☒	For hierarchical and complex designs, identification of the appropriate level for tests and full reporting of outcomes
☐	☒	Estimates of effect sizes (e.g. Cohen's *d*, Pearson's *r*), indicating how they were calculated

Our web collection on statistics for biologists contains articles on many of the points above.

Software and code

Policy information about availability of computer code

Data collection	All data analyzed within this manuscript are publicly available. No additional software was used for the data collection process.
Data analysis	DeepTalk analysis was performed using Python(Version 3.8.0) package DeepTalk_ST(Version 0.0.2) (available at https://github.com/JiangBioLab/DeepTalk). Benchmarked methods were performed using sotware packages squidpy (Version 1.2.3) and Scanpy(Version 1.9.6) . Full benchmark scripts and environment configuration also be found at https://github.com/JiangBioLab/DeepTalk.<br>We compared the performance of 9 integration methods for predicting the spatial distribution of undetected transcripts: DeepTalk_ST (Version 0.0.2) , gimVI(Version 0.8.0b0), spaGE(no version), Tangram(Version 1.0.0), Seurat(Version 3.6.3), SpaOTsc(Version 0.2), LIGER (Version 0.5.0), novospaRc(Version0.4.3), stPlus(Version 0.0.6). We compared the performance of 8 integration methods for predicting the celltype composition of spots: DeepTalk_ST(Version 0.0.2), Cel2location(Version 0.6a0), DestVI(Version 0.14.4), SPOTlight(Version 0.1.7), SpatialDWLS(version 1.0.4), Tangram(Version 1.0.0), RCTD(version1.2.0), stereoscope(Version 0.14.4).<br>We compared the performance of 16 cell-cell communication inference methods for predicting cell-cell communications: DeepTalk_ST (Version 0.0.2) , COMMOT(Version 0.0.3), CellCall(Version 0.0.0.9000), CellChat(Version 1.0.0), CellChatDB v2(Version 2.0.0), CellPhoneDB (Version 2.1.7), CellPhoneDB v3(Version 3.0.1), Connectome(Version 1.0.1), Giotto(Version 1.0.4), ICELLNET(Version 0.99.3), iTALK(Version 0.1.0), NicheNet(Version 1.0.0), SingleCellSignalR(Version 1.4.0), stLearn(Version 0.4.7), NICHES(Version 1.0.0), scriabin(Version 0.0.0.9000).

For manuscripts utilizing custom algorithms or software that are central to the research but not yet described in published literature, software must be made available to editors and reviewers. We strongly encourage code deposition in a community repository (e.g. GitHub). See the Nature Portfolio guidelines for submitting code & software for further information.

Data

Policy information about availability of data

All manuscripts must include a data availability statement. This statement should provide the following information, where applicable:

- Accession codes, unique identifiers, or web links for publicly available datasets
- A description of any restrictions on data availability
- For clinical datasets or third party data, please ensure that the statement adheres to our policy

This study made use of publicly available datasets. The detailed information of 45 paired spatial transcriptomics and scRNA-seq datasets, along with 32 simulated datasets for assessing the effectiveness of the integration method, were retrieved from https://drive.google.com/drive/folders/1pHmE9cg_tMcouV1LFJFtbyBJNp7oQo9J?usp=sharing. MERFISH VISp data and Smart-Seq2 VISp snRNA-seq data were available at http://github.com/spacetx-spacejam/data. 10x Genomics Visium Fluorescent dataset is available from https://support.10xgenomics.com/spatial-gene-expression/datasets and adult mouse cortical region scRNA-seq data were obtained through GEO under accession number GSE115746. The scRNA-seq and ST data of the human PDAC data were obtained through GEO under accession number GSE111672. Source data for the main figures are provided with this paper. Source data are provided with this paper.

Research involving human participants, their data, or biological material

Policy information about studies with human participants or human data. See also policy information about sex, gender (identity/presentation), and sexual orientation and race, ethnicity and racism.

Reporting on sex and gender	None
Reporting on race, ethnicity, or other socially relevant groupings	None
Population characteristics	None
Recruitment	None
Ethics oversight	None

Note that full information on the approval of the study protocol must also be provided in the manuscript.

Field-specific reporting

Please select the one below that is the best fit for your research. If you are not sure, read the appropriate sections before making your selection.

☒ Life sciences ☐ Behavioural & social sciences ☐ Ecological, evolutionary & environmental sciences

For a reference copy of the document with all sections, see nature.com/documents/nr-reporting-summary-flat.pdf

Life sciences study design

All studies must disclose on these points even when the disclosure is negative.

Sample size

No sample size chosen was performed. All data used in this manuscript were taken from public resources and used to demonstrate the ability of DeepTalk.

We used 45 paired spatial transcriptomics and scRNA-seq datasets from published studies for integrating spatial transcriptomics and scRNA-seq data. The details of these datasets are listed as follows:

Dataset 1 has 8425 spots in spatial transcriptomics data and 4651 cells in scRNA-seq data;
Dataset 2 has 175 spots in spatial transcriptomics data and 9991 cells in scRNA-seq data;
Dataset 3 has 3585 spots in spatial transcriptomics data and 8596 cells in scRNA-seq data;
Dataset 4 has 524 spots in spatial transcriptomics data and 14249 cells in scRNA-seq data;
Dataset 5 has 2050 spots in spatial transcriptomics data and 31217 cells in scRNA-seq data;
Dataset 6 has 4975 spots in spatial transcriptomics data and 31299 cells in scRNA-seq data;
Dataset 7 has 645 spots in spatial transcriptomics data and 9234 cells in scRNA-seq data;
Dataset 8 has 6963 spots in spatial transcriptomics data and 7240 cells in scRNA-seq data;
Dataset 9 has 2399 spots in spatial transcriptomics data and 14249 cells in scRNA-seq data;
Dataset 10 has 1549 spots in spatial transcriptomics data and 14249 cells in scRNA-seq data;
Dataset 11 has 1380 spots in spatial transcriptomics data and 7737 cells in scRNA-seq data;
Dataset 12 has 6000 spots in spatial transcriptomics data and 15928 cells in scRNA-seq data;
Dataset 13 has 6000 spots in spatial transcriptomics data and 14249 cells in scRNA-seq data;
Dataset 14 has 3039 spots in spatial transcriptomics data and 1297 cells in scRNA-seq data;
Dataset 15 has 3405 spots in spatial transcriptomics data and 5613 cells in scRNA-seq data;
Dataset 16 has 11426 spots in spatial transcriptomics data and 14249 cells in scRNA-seq data;
Dataset 17 has 1154 spots in spatial transcriptomics data and 14249 cells in scRNA-seq data;

Dataset 18 has 982 spots in spatial transcriptomics data and 4748 cells in scRNA-seq data;
Dataset 19 has 995 spots in spatial transcriptomics data and 4816 cells in scRNA-seq data;
Dataset 20 has 4784 spots in spatial transcriptomics data and 6178 cells in scRNA-seq data;
Dataset 21 has 4895 spots in spatial transcriptomics data and 6178 cells in scRNA-seq data;
Dataset 22 has 2432 spots in spatial transcriptomics data and 6178 cells in scRNA-seq data;
Dataset 23 has 1211 spots in spatial transcriptomics data and 6178 cells in scRNA-seq data;
Dataset 24 has 1162 spots in spatial transcriptomics data and 6178 cells in scRNA-seq data;
Dataset 25 has 1127 spots in spatial transcriptomics data and 6178 cells in scRNA-seq data;
Dataset 26 has 2425 spots in spatial transcriptomics data and 1911 cells in scRNA-seq data;
Dataset 27 has 198 spots in spatial transcriptomics data and 3415 cells in scRNA-seq data;
Dataset 28 has 277 spots in spatial transcriptomics data and 4740 cells in scRNA-seq data;
Dataset 29 has 1835 spots in spatial transcriptomics data and 10872 cells in scRNA-seq data;
Dataset 30 has 2064 spots in spatial transcriptomics data and 13600 cells in scRNA-seq data;
Dataset 31 has 3805 spots in spatial transcriptomics data and 8798 cells in scRNA-seq data;
Dataset 32 has 3024 spots in spatial transcriptomics data and 3512 cells in scRNA-seq data;
Dataset 33 has 3024 spots in spatial transcriptomics data and 8653 cells in scRNA-seq data;
Dataset 34 has 1888 spots in spatial transcriptomics data and 8346 cells in scRNA-seq data;
Dataset 35 has 744 spots in spatial transcriptomics data and 4561 cells in scRNA-seq data;
Dataset 36 has 369 spots in spatial transcriptomics data and 7268 cells in scRNA-seq data;
Dataset 37 has 2125 spots in spatial transcriptomics data and 7185 cells in scRNA-seq data;
Dataset 38 has 2669 spots in spatial transcriptomics data and 3499 cells in scRNA-seq data;
Dataset 39 has 9852 spots in spatial transcriptomics data and 3499 cells in scRNA-seq data;
Dataset 40 has 41674 spots in spatial transcriptomics data and 26252 cells in scRNA-seq data;
Dataset 41 has 6000 spots in spatial transcriptomics data and 10000 cells in scRNA-seq data;
Dataset 42 has 1145 spots in spatial transcriptomics data and 48164 cells in scRNA-seq data;
Dataset 43 has 604 spots in spatial transcriptomics data and 15095 cells in scRNA-seq data;
Dataset 44 has 6000 spots in spatial transcriptomics data and 10259 cells in scRNA-seq data;
Dataset 45 has 2177 spots in spatial transcriptomics data and 981 cells in scRNA-seq data;
We used 3 paired spatial transcriptomics and scRNA-seq datasets from published studies for the inference of cell-cell communication. The details of these datasets are listed as follows:
2399 cells in MERFISH spatial transcriptomics dataset and 11759 cells in SMART-Seq2 snRNA-seq dataset;
324 spots in 10X Visium spatial transcriptomics dataset and 21697 cells in 10X Visium snRNA-seq dataset;
426 spots in human pancreatic ductal adenocarcinoma (PDAC) ST dataset and 1926 cells in PDAC scRNA-seq dataset.

Data exclusions

No data were excluded from the analysis. Filtering and quality control of single-cell RNA-seq data is described in the Methods.

Replication

All computational experiments and environment configuration files are provided to ensure reproducibility. All experiments were reproduced a minimum of three times. All attempts at replication were successful and can be performed independently.
To make sure that the experimental findings are reproducible, we (1) verified the performance of the 9 integration methods for predicting the spatial distribution of undetected transcripts on 45 paired datasets, (2) verified the performance of the 8 integration methods for predicting the cell type composition of spots on 32 paired simulated datasets, (3) verified the performance of of the 16 cell-cell communication inference methods for predicting the cell-cell communications on 3 paired datasets.

Randomization

This is not relevant to our study because we reanalyzed publicly available data. Each dataset was analysed independently, and the evaluation of our method compared to the state-of-the art methods did not require any experimental groups.

Blinding

All computational methods were blinded to ground truth pairing information during performance evaluation.

Reporting for specific materials, systems and methods

We require information from authors about some types of materials, experimental systems and methods used in many studies. Here, indicate whether each material, system or method listed is relevant to your study. If you are not sure if a list item applies to your research, read the appropriate section before selecting a response.

Materials & experimental systems

n/a	Involved in the study
☒	☐ Antibodies
☒	☐ Eukaryotic cell lines
☒	☐ Palaeontology and archaeology
☒	☐ Animals and other organisms
☒	☐ Clinical data
☒	☐ Dual use research of concern
☒	☐ Plants

Methods

n/a	Involved in the study
☒	☐ ChIP-seq
☒	☐ Flow cytometry
☒	☐ MRI-based neuroimaging

Plants

Seed stocks	This study does not involve plant information.
Novel plant genotypes	This study does not involve plant information.
Authentication	This study does not involve plant information.
